# Wheat Biofortification: Utilizing Natural Genetic Diversity, Genome-Wide Association Mapping, Genomic Selection, and Genome Editing Technologies

**DOI:** 10.3389/fnut.2022.826131

**Published:** 2022-07-12

**Authors:** Om Prakash Gupta, Amit Kumar Singh, Archana Singh, Gyanendra Pratap Singh, Kailash C. Bansal, Swapan K. Datta

**Affiliations:** ^1^ICAR-Indian Institute of Wheat and Barley Research, Karnal, India; ^2^ICAR-National Bureau of Plant Genetic Resources, New Delhi, India; ^3^Department of Botany, Hansraj College, University of Delhi, New Delhi, India; ^4^National Academy of Agricultural Sciences, New Delhi, India; ^5^Department of Botany, University of Calcutta, Kolkata, India

**Keywords:** micronutrients, hidden hunger, phytate, QTLs, genome editing

## Abstract

Alleviating micronutrients associated problems in children below five years and women of childbearing age, remains a significant challenge, especially in resource-poor nations. One of the most important staple food crops, wheat attracts the highest global research priority for micronutrient (Fe, Zn, Se, and Ca) biofortification. Wild relatives and cultivated species of wheat possess significant natural genetic variability for these micronutrients, which has successfully been utilized for breeding micronutrient dense wheat varieties. This has enabled the release of 40 biofortified wheat cultivars for commercial cultivation in different countries, including India, Bangladesh, Pakistan, Bolivia, Mexico and Nepal. In this review, we have systematically analyzed the current understanding of availability and utilization of natural genetic variations for grain micronutrients among cultivated and wild relatives, QTLs/genes and different genomic regions regulating the accumulation of micronutrients, and the status of micronutrient biofortified wheat varieties released for commercial cultivation across the globe. In addition, we have also discussed the potential implications of emerging technologies such as genome editing to improve the micronutrient content and their bioavailability in wheat.

## Introduction

Micronutrient deficiency also known as “hidden hunger,” is one of the major global health problems afflicting more than 2 billion people globally (1). Micronutrient deficiency is common among population groups dependent on a single cereal (rice, wheat or maize) diet. These populations lack access to an adequate quantity of fruits, vegetables, dairy products, meats, etc. that are rich in essential minerals and vitamins. Globally, the human populations in Sub-Saharan Africa and South Asia bear the most significant burden of these micronutrient deficiencies (2). Among the micronutrient deficiencies, Iron (Fe), Zinc (Zn), Iodine (I), and Vitamin A deficiencies are the most widespread. Other micronutrient deficiencies include Calcium (Ca) and selenium (Se) that are relatively less widespread but could become more prevalent in the future if not addressed now. The daily intake of these micronutrients recommended dietary allowance (RDA) is vital to sustaining life as they are required for the proper physical and cognitive development, disease prevention, and overall human well-being. Children less than five years of age, pregnant women, and lactating women are amongst the most vulnerable group to such micronutrient deficiencies.

Fe is required for several processes in the human body, including oxygen transport, electron transport, and DNA synthesis (3). For example, it is a component of oxygen transport proteins, such as hemoglobin and myoglobin. Moreover, Fe is also required for many proteins and enzymes involved in the energy generation, synthesis of neurotransmitters, and proper functioning of the immune system (4). Deficiency of Fe is the leading cause of anemia; nearly 30% of the women of reproductive age (14-59 years of age) and 40% of the children under five years suffer from anemia, globally (5). Similarly, Zn is also a trace element required for proper growth and maintenance of the human body. It acts as an essential cofactor for over 300 enzymes that are involved in vital processes, including cell proliferation, healing of wounds, blood clotting, etc. (6, 7). Zn deficiency has also been associated with increased diarrheal diseases and acute respiratory infections in children under five years of age and is a critical factor contributing to disease burden in developing countries (8). In Africa, 58% of child deaths are estimated to be due to Zn deficiency (9). Se is another important essential trace element needed for a robust immune system, thyroid function and reproduction. It is an essential component of selenoproteins, which act as potent antioxidant protecting cellular components from free radicals (10). Globally, an estimated 1 billion population suffers from Se deficiency, and this number is expected to rise in coming decades, necessitating designing strategies to enhance its dietary intake (11). Ca has also been regarded as one of the essential micronutrients required for the proper growth and development of the human body. It is required for strong bones and teeth and is also involved in many fundamental processes, such as blood coagulation, muscular function, hormonal secretions, nerve impulse transmission, etc. (12). Its deficiency can cause many problems, such as rickets in children, and osteoporosis and osteopenia in adults.

For curbing hidden hunger in the developing world, increasing the content *via* biofortification *vis-à-vis* increasing the bioavailability of both Fe and Zn are the two major approaches. For the first approach i.e., biofortification, significant variation in the level of Fe (up to 88 mg kg^–1^) and Zn (14 to 190 mg kg^–1^) has been reported among wild wheat, especially wild emmer wheat (*Triticum turgidum* ssp. *Dicoccoides*) (13), which can be efficiently utilized by wheat breeders to transfer in the background of high yielding and disease resistant hexaploid wheat genotypes. A breeding target of > 59 μg g^–1^ Fe, and 38 μg g^–1^ Zn in wheat grains (14) against the baseline level of 30 μg g^–1^ Fe, and 25 μg g^–1^ Zn would be sufficient to meet the 30–40% of the average daily requirement of an adult. However, bioavailability of Fe and Zn in wheat is greatly limited due to the presence of phytic acid (PA, 0.4–2.0%), an anti-nutrient (15, 16). [PA]:[Fe and Zn] ratios are very vital in determining the potential bioavailability of the micronutrients and are inversely proportional i.e., higher the molar ratio, lesser the bioavailability and *vice-versa*. For [PA]:[Fe], the ratio should be < 1 (preferably < 0.4) to significantly improve Fe absorption (17), while for [PA]:[Zn] ratios of < 5, 5 to 15, and > 15 have been associated with high (50%), moderate (30%) and low (15%) Zn bioavailability, respectively (18). Therefore, it is desired that wheat genotypes be developed with suitable [PA]:[Fe and Zn] ratios for optimum bioavailability of Fe and Zn to humans and animals.

Generally, three major strategies i.e., dietary diversification, food fortification, and food supplementation were developed to address the problem of micronutrient deficiencies (19). Among these, dietary diversification focuses on modifying food consumption patterns at the individual household level, such as increasing the intake of more nutritious diets like fruits, vegetables, animal foods, etc. However, dietary diversification is not possible in many parts of the world due to poor socio-economic conditions and ethnic dietary choices. Other alternatives like food fortification and micronutrient supplementation for specific life stages and age groups can be considered stopgap measures for tackling micronutrient deficiencies. However, these strategies cannot provide a long-term sustainable solution for nutrient deficiencies in low and middle-income countries. These countries have a large population that lives in extreme poverty and does not have both physical as well as economic access to the adequate quantity of nutritious foods. Further, they cannot afford fortified food products or food supplements (20). Moreover, setting up the infrastructure to develop and distribute fortified foods or even food supplements would require a considerable investment that underdeveloped countries cannot afford. Agro-system diversification can assist local populations to expand their food baskets and solve the problem of micronutrient deficiencies, but it cannot be widely adopted in underdeveloped countries due to small landholdings. Therefore, in the past two decades, a greater emphasis has been laid on biofortification, which refers to increasing the bioavailable nutrient content of food crops either through conventional plant breeding or transgenic approaches. Biofortification is considered the most effective and sustainable approach for addressing the micronutrient deficiencies related problems in humans (21). A recent publication “Wheat and Barley Grain Biofortification” (Elsevier, United Kingdom), would serve as an important ready reckoner for different domains of wheat biofortification (22).

Wheat supplies approximately 20% of the human population’s total calories and protein intake worldwide. However, most of the commercial wheat cultivars grown across the world are deficient or have suboptimal levels of micronutrients. It is mainly due to the greater focus of national wheat breeding programs on increasing yield, which has resulted in the erosion of grain minerals and protein contents in improved varieties. For the development of nutrient-dense wheat varieties, the primary prerequisite is to identify donor lines with high concentrations of the targeted micronutrients. Therefore, the need of the hour is to explore natural genetic diversity among the landraces and wild wheat species for grain mineral content and utilize them in the breeding programs for developing biofortified varieties. In 2003, a program in this direction was initiated at CIMMYT, with the support from HarvestPlus. The breeding materials developed under this program have contributed to the development of few biofortified cultivars with higher grain Zn and Fe concentrations in India, Pakistan, Nepal, Mexico, and Bangladesh (23).

In wheat, conducting molecular studies, especially cloning of genes for any target trait, is considered a very challenging task due to the large and complex genome organization. Therefore, it took many years to clone a grain protein content (GPC) gene (*Gpc-B1*) from an accession of wild emmer wheat (*Triticum turgidum*, *dicoccoides*) (24). Even for the qualitative traits, such as disease resistance, which are generally controlled by a single major gene, cloning the resistance locus has never been easier due to the lack of information on the whole genome-level sequence as well as fully sequenced genes. However, in recent years, the availability of genomic resources such as the gold standard reference sequence of hexaploid wheat (25), reference genome sequences of its progenitor species (26, 27), transcriptome landscape of different tissues of wheat (28–31), single nucleotide polymorphism (SNP) genotyping arrays and genotyping by sequencing (GBS) methods (32) has led to a revolution in the field of wheat genomics. These tools have made it relatively easier to fine map and clone genes of essential traits in cultivated and wild wheat species (33). Further, in recent years, genome-wide association study (GWAS), which uses diverse germplasm lines or multiparent populations, has developed as a powerful tool for high-resolution trait mapping in crops and can be used to map nutritional quality traits using diverse association panels constituted from landraces, synthetics and wild species (34–36). Furthermore, recently, genome editing technologies, including prime editing and base editing have become promising targeted mutagenesis tools for crop improvement (37, 38). The first report of gene editing in wheat was the development of the targeted knockout for the *Mlo* gene that confers resistance against powdery mildew pathogen, *Blumeria graminis f*.sp. *tritici* (39). Since then, there are many reports of gene editing in wheat targeting genes associated with various agronomical and quality traits (40). Recently, nano-technology has also been explored for micronutrient biofortification in wheat. Khan et al. (41) have extensively reviewed the status of nano formulation-based wheat biofortification with a critical analysis of its merits and demerits.

In the present review, we have discussed the current state of knowledge on the existing natural genetic variations for micronutrients content among cultivated and wild wheat germplasm, genes and genomic regions controlling the micronutrient traits, current status of biofortified wheat varieties released for commercial cultivation around the world and potential applications of genome editing tools in the improvement of nutritional quality traits in wheat.

## Exploring Natural Genetic Variation for Grain Micronutrients in Wheat and Its Wild Relatives

Understanding the extent and magnitude of natural genetic variations for various essential nutrients in wheat and its wild species is critical for improving these traits through classical and modern breeding tools. In this context, extensive screening of germplasm collection of wheat and its wild species conserved in genebanks of national and international institutions can facilitate the discovery of novel germplasm donors for various essential nutrients. These donor germplasms can be further exploited in the breeding programs for the development of biofortified wheat varieties. Over the past two decades, several studies have explored cultivated and wild wheat germplasm for variations in grain micronutrient contents. The key findings of some of these studies are briefly presented below.

### Iron (Fe), Zinc (Zn), Selenium (Se) and Calcium (Ca) Content

Several studies have reported variation in grain Fe and Zn concentrations of bread wheat cultivars, landraces and wild wheat (42–44). Generally, breeding lines and cultivars have low genetic variation for grain Fe and Zn concentrations compared to landraces, cultivated wheat progenitors and related wild wheat species (unadopted wheat) (45). Several studies show a negative correlation between mineral concentrations and yield, implying that increase in grain yield of wheat varieties was accompanied by a significant decrease in their grain mineral content (45, 46). Evaluation of eighty Iranian wheat cultivars bred over a period of 70 years revealed a significant decrease in grain Fe and Zn concentrations that ranged from 63.56 to 102.19 and 31.65 to 54.06 mg/kg, respectively (46). On the other hand, a wide range of variations for grain Fe and Zn concentrations have been reported in landraces and other unadopted germplasms (43, 44, 47, 48). Qury et al. (43) analyzed a diverse wheat genotype panel comprising of French landraces, elite breeding lines, modern varieties and a set of worldwide germplasm collection which showed variation in grain Zn and Fe concentrations ranging from 15 to 35 mg/kg, and 20 to 60 mg/kg, respectively. However, some unadopted lines of this panel had Fe and Zn concentrations as high as 88 and 43 mg/kg, respectively, which can be exploited to improve the wheat cultivar’s mineral concentrations. Another study on fifty landraces and ten varieties revealed higher Fe (24.93 to 66.51 mg/kg) and Zn (18.68 to 38.66 mg/kg) concentrations in landraces as compared to commercial cultivars (48). Recently, novel sources of variation for whole-grain Fe and Zn concentrations were identified in a panel of 245 diverse bread wheat lines derived from crosses between landraces of Watkin collections with a United Kingdom wheat cultivar Paragon (49). Further, the above studies have found that wide variation in grain Fe and Zn concentrations of wheat genotypes across different studies may not be solely due to genotypic differences since environment and soil nutrient status are known to significantly affect these traits, so promising Fe and Zn rich lines identified in these studies must be validated in multilocation trails.

Contrary to widely cultivated wheat, the primary and secondary gene pool of wheat such as *Triticum monococcum*, *Triticum boeoticum*, *T. turgidum dicoccoides*, *Aegilops tauschii, T. spelta* and *Triticum polonicum* are reported to contain wider variation for grain Fe and Zn concentrations (34, 45, 50–52). Among these species, *T. turgidum* ssp. *dicoccoides* is considered the most promising donor for the grain Fe and Zn content. Cakmak et al. (53) screened a large number of accessions of several wild wheat species and their relatives for grain Fe and Zn concentrations and observed unique variations among the *T. dicoccoides* accessions, which ranged from 14 to 190 mg/kg for Zn and from 15 to 109 mg/kg for Fe. Studies have also identified some accessions with high grain Fe and Zn concentrations in other species as well. Tiwari et al. (52) screened a large number of accessions of *T. boeoticum* and found one accession (pau5088) with higher levels of grain Fe and Zn concentration, 40.1 and 44.6 mg/kg, respectively.

Additionally, some non- progenitor, wild wheat species such as S, U and M genomes have been reported to contain 3-4 times more Fe and Zn than cultivated hexaploid and tetraploid species (51). Velu et al. (54) reported wide genetic variability for Fe and Zn concentration among the introgression lines derived from cultivated species’ crosses with wild wheat rich in grain Fe and Zn content. In addition to wild species of wheat, a few non-*Triticum* species such as rye and *Leymus* spp are also rich in mineral nutrient contents. The wheat-alien introgression lines derived from the crosses with *Leymus racemosus*, and also those carrying introgression of 2R, 3R chromosomes of rye contain a high level of Fe and Zn (55).

There are a few reports on the genetic variability of Se concentration in cultivated wheat and its wild relatives as compared to that of Fe and Zn (45, 56, 57). Lyons et al. (56) analyzed the Se concentration of ancestral and wild relatives of wheat, landraces, population, and cultivars grown in Mexico and Australia. The grain Se concentration of this set ranged from 5–720 μg/kg; however, much of this variation was attributed to variation in soil Se content across the locations. Nevertheless, they reported higher variation in diploid species *Ae. tauschi* and rye. Similarly, Zhao et al. (45) also reported limited genetic variability for grain Se concentration in commercial wheat cultivars. Apart from Fe, Zn, and Se, there are very limited genetic variability studies for Ca content in wheat. A study on Indian and Iranian wheat lines showed phenotypic variability for grain Ca content in the range of 104.3 to 663.5 mg/kg (58). Another study that analyzed a diverse panel of 353 wheat varieties, including winter and spring wheat varieties, reported wide variations for grain Ca content ranging from 288.2 to 647.5 mg/kg. Nirvana, a wheat variety from France, had a very high concentration of grain Ca (647.5 mg/kg DW) (59). The wide variability for grain Ca content in the above two studies suggest ample scope for developing biofortified Ca wheat varieties.

### Phytate Content

The phytic acid content in wheat grain can significantly affect the bioavailability of minerals such as Fe and Zn during digestion because of their strong ability to bind to metals. Therefore, wheat genotypes with low phytate and high mineral concentration could be immensely useful in breeding programs that aim to develop biofortified verities for essential mineral nutrients. Many studies have analyzed the variability of phytate content among wheat cultivars and germplasm lines (60–62). The analysis of phytic acid content of a set of 65 bread varieties of Pakistan showed variation in the range 0.706–1.113% (60). Another comprehensive study on the collection of global durum cultivars identified a 2-fold variation in phytic acid content ranging between 0.462 to 0.952% (62). Contrary, many other studies have identified higher values for phytic acids (more than 1%) in durum genotypes, which might be attributed to G × E effects (61, 63). The low phytate genotypes identified in various studies may be potentially used as parents for developing wheat varieties with enhanced bioavailable Fe and Zn levels.

## Genomic Regions/Genes Controlling Micronutrients in Wheat and Its Wild Relatives

Advancements in genomics, especially the availability of high-throughput genotyping assays such as whole genome re-sequencing, GBS, SNP arrays, etc., have made it easier to perform trait mapping in plant species like wheat with a large and complex genome. Both bi-parental and association mapping approaches have facilitated identifying several QTLs/genomic regions controlling grain minerals content. A brief description of the genomic regions/genes identified in the various studies is presented below.

### Genomic Regions/Genes Associated With Grain Fe, Zn, Ca, and Se

In the past two decades, several studies have reported QTLs/candidate genes for grain Fe and Zn concentrations in wheat and its wild species using both QTL and association mapping methods (Table 1). Expectedly, most QTLs for Fe and Zn were identified from wild wheat and their relatives because there is a minimal variability for both these minerals in cultivated wheat germplasm. Tiwari et al. (52) were the first to report genetic mapping of grain Fe and Zn using an interspecific mapping population derived from the cross of *T. boeoticum* accession pau5088 (high grain Fe and Zn concentration) with *T. monococcum* accession pau14087. They identified two QTLs for grain Fe and one QTL for Zn. After that, many other studies have employed bi-parental mapping approach and identified QTLs for grain Fe and Zn concentrations on various chromosomes of wheat and its wild relatives (64–69). In the past few years, the association mapping approach has also facilitated identifying genomic regions/QTLs associated with grain Fe and Zn concentrations in diverse association panels constituted using diverse genotypes, including synthetic hexaploid wheat, advanced breeding lines, landraces and cultivars etc. However, most of the reported grain Fe and Zn QTLs have not been found stable across various locations suggesting profound effects of environment and genotype X environment on both these traits. Further, many identified regions have minor effects on grain Fe and Zn concentrations. Therefore, only the significant QTLs for Fe and Zinc identified in various studies should be focused on improving cultivated wheat’s mineral contents. Some of the significant QTLs for zinc concentration has been identified on chromosome 1B, 2B, 5A, 1B, 6B (65, 70). Moreover, some studies have identified common genomic regions for grain Fe and Zn concentrations, and even some are also associated with other valuable traits such as thousand-grain weight, protein etc. (65, 71). Tiwari et al. (65) had identified two major QTL for grain zinc concentration on 1B and 2B; of these, the QTL on 2B was colocalized with the QTL for grain Fe concentration. Similarly, a significant QTL for grain Zn on 2B co-located with the QTL for grain Fe concentration (66). These studies suggest that simultaneous improvement of both traits is possible using MAS. Compared to Fe and Zn, the QTL mapping studies for grain Se concentration in wheat are rare. A total of five QTLs for Se content were identified on chromosome 3D, 4A, 4D, 5B, and 7D, using two different RIL populations (72). Moreover, in a recent study, Wang et al. (73) identified nine Se concentration QTLs in a mapping population derived from the cross of winter wheat cultivars Tainong18 and Linmai6.

**TABLE 1:** Genomic regions/QTLs identified for grain Zn and Fe concentrations in cultivated and wild wheat using biparental and association analysis methods.

Mapping approach	Parentage/association panel	Chromosome	Number of QTLs/genomic regions		Phenotypic variance (%)		References
			Fe	Zn	Fe	Zn	
QTL mapping	*T. boeoticum* (pau5088)x *T. monococcum* (pau14087)	Fe: 2A, 7A (2 QTLs) Zn: 7A (2 QTLs)	3	2	7.0–12.6	9.0–18.0	Tiwari et al. (52)
QTL mapping	Durum wheat (cv. Langdon) X wild emmer (accession G18-16).	Fe: 2A (2 QTLs), 2B, 3A, 3B, 4B 5A, 6A, 6B, 7A, 7B Zn: 2A (2 QTLs), 5A, 6B, 7A, 7B	11	6	2–18	1–23	Peleg et al. (64)
QTL mapping	Xiaoyan 54 and Jing 411	Fe: 2B, 5A, 6A Zn: 5A, 2A, 4B	3	3	3.27–10.78	4.23–9.05	Xu et al. (70)
QTL mapping	PBW343X Kenya Swara	Zn: 1B, 2B, 3A, 4A, 5B	–	5		10.0–15.0	Hao et al. (71)
QTL mapping	Berkut X Krichauff	Fe: 1B Zn:1B,2B	1	2	22.2	23.1–35.90	Tiwari et al. (65)
QTL mapping	Two mapping populations were used: Saricanak98 X MM5/4 Adana99 × 70,711	Fe: 1B, 2A, 2B (2 QTLs), 3A, 6B, 7B Zn: 1B, 1D, 2B, 3A, 3D, 6A, 6B, 7A (2 QTLs), 7B	7	10	9.0–17.	9.00–31.0	Velu et al. (66)
Association mapping	167 *Ae. tauschii*	Fe: 1D, 2D, 3D, 4D, 7D Zn: 2D, 4D, 6D, 7D	5	4	–	–	Arora et al. (114)
QTL mapping	WH542 X a synthetic derivative [*Triticum dicoccon* PI94624/*Aegilops tauschii* (409)//BCN].	Fe: 6D, 7D (2 QTLs) Zn: 1D, 3B, 2D (2 QTLs), 7D (2 QTLs)	3	6	5.61–42.12	5.05–13.07	Krishnappa et al. (69)
Association mapping	369 European elite wheat varieties	Zn: 2A, 3A, 3B, 4A, 4D, 5A, 5B, 5D, 6D, 7A, 7B, 7D	–	40	–	2.5–5.2	Alomari et al. (59)
Association mapping	123 synthetic hexaploid wheat derived from cross *Triticum turgidum* L. × *Aegilops tauschii* Coss.	Fe: 1A (2 QTLs), 3A Zn: 1A, 2A (2 QTLs, 3A (2 QTLs), 3B (3 QTLs), 4A, 4B, 5A (2 QTLs), 6B	3	13	11.2–13.2	1.8–14.1	Bhatta et al. (115)
QTL mapping	Roelfs F2007X Hong Hua Mai/./Blouk #1	Fe: 1A, 2A, 3B, 3D, 4B, 5A, 6B (2 QTLs) Zn: 1B, 2B, 3A, 3B, 3D, 4B, 5A (2 QTLs), 6B, 7A	9	10	2.10–14.56	2.71–14.22	Liu et al. (68)
Association mapping	HarvestPlus Association Mapping panel consisted of 330 wheat lines.	Zn: 1A, 2A (10 QTLs), 2B (11 QTLs), 2D (2 QTLs), 5A (2 QTLs), 6B (2 QTLs), 6D, 7B (7QTLs), 7D	–	39	–	5–10.5	Velu et al. (34)
QTL mapping	WH542 X a synthetic derivative [*Triticum dicoccon* PI94624/*Aegilops tauschii* (409)//BCN].	Fe: 6D, 7D (2 QTLs) Zn: 3B, 1D, 2D (2 QTLs), 7D (2 QTLs)	3	6	5.01–13.07	5.61–42.13	Krishnappa et al. (69)
QTL mapping	Kachu × Zinc-Shakti	Fe: 1B, 1D, 2A, 6A Zn: 1B (2QTLs), 1D, 2A, 2B, 5A, 6B, 7D (2 QTLs)	4	9	3.1–12.3	3.3–10.3	Rathan et al. (116)
Association mapping	205 wheat genotypes comprising cultivars, landraces, and breeding lines	Zn: 2B, 3B, 4B, 7B, 7A Fe: 5A, 6B, 7B, 7D	20	16	8.07–16.23	7.94–12.12	Wang et al. (117)

In contrast to grain Fe and Zn content, there is very limited information on genomic regions/QTLs for grain Ca accumulation. A total of 9 QTLs for grain Ca were reported in the RIL mapping population derived from durum and wild emmer wheat (64). In another study, association mapping using a diverse panel of European wheat accessions identified genomic regions for grain Ca accumulation on all the wheat chromosomes except 3D, 4B, and 4D (59). Recently, Alomari et al. (74) identified a major genomic region for grain Ca on the long arm of 5A, which overlapped with gene *TraesCS5A02G542600* that encoded for a transmembrane protein.

## Integration of Gwas With Multi-Omics Data to Accelerate the Discovery of Candidate Genes for Biofortification Traits From Wheat Germplasm

**FIGURE 1:**
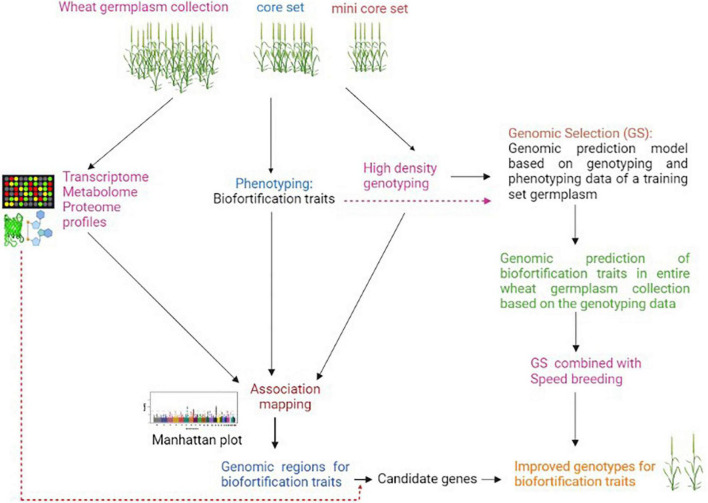
Scheme for integration of omics and genomic selection approaches for accelerating improvement of biofortification traits in wheat.

Biofortification traits have complex regulations and are governed by many QTLs/genes, which are significantly affected by environment and genotype-environment interactions (75). The expression of some biofortification traits such as grain mineral content involves many processes such as mineral absorption, translocation, redistribution, and re-mobilization to sink, and each of these processes is controlled by many genes. This makes genetic dissection of such traits challenging by utilizing any single genetic or molecular analysis approach (69, 76). The conventional GWAS approach identifies a large number of genomic regions/QTLs that can not be directly utilized in a breeding program (34). Moreover, GWAS does not go beyond simple marker-trait correlation with no proof of causality; therefore, this approach alone may not provide insights on the functional basis of variation in biofortification traits. The above two limitations of GWAS can be overcome by incorporating functionome (multi-omics) data (77). In the past decade, significant technological improvements in the field of “omics” have made it feasible to generate large-scale omics data such as transcriptome, proteome, and metabolome, etc., from a large number of samples at a low cost (78). Integration of GWAS with various multi-omics data would enable a system-level understanding of biofortification traits and has great potential to precisely pinpoint the actual causal variant/candidate gene. There can be two approaches for integrating multi-omics data in GWAS analysis; 1) GWAS is independently performed using biofortification trait profiling data as well as associated omics data i.e., gene expression, proteome and metabolome data of association panel genotypes, and then marker-trait associations results are integrated to interpret pathways and identify causal variant/candidate genes associated with the traits; 2) GWAS is performed only using genome-wide DNA markers and biofortification traits, and then expression, proteome, and metabolite profiling data generated from a few contrasting genotypes of the association panel are mapped to genomic regions associated with the targeted trait to identify the candidate genes (Figure 1). The integration of functionome data in GWAS analysis of biofortification traits may not only help identify causal variants responsible for these traits but would also enable their comprehensive understanding at the cellular, biochemical and molecular levels.

## Implementation of Genomic Selection and Speed Breeding Has the Potential to Accelerate Wheat Biofortification

In recent years, with the availability of high throughput and cost-effective genotyping assays, genomic selection (GS) has emerged as a promising genomics-based tool for the improvement of complex traits in crops (79). In GS, the selection of elite genotypes is made using genomic estimated breeding values (GEBVs), which consider all marker effects across the genome. GS enhances breeding efficiency for quantitative traits by reducing breeding cycle duration and selection gain per unit time^1^. This approach has great potential for improving the quality traits with low genetic variance (80). Joukhadar et al. (81) have discussed in detail the potential application of GS in the biofortification of spring wheat. There are already some reports on genomic predictions of micronutrient traits in crops (82–84). Owens et al. (82) were among the first to estimate genomic prediction for a biofortification trait in a crop. They predicted pro-vitamin A content in maize using genome-wide as well as carotenoid pathway-based markers and identified a small number of candidate genes that can be targeted for conversion of elite genotype with low carotenoid content to one that has an orange color grain with higher levels of high pro-vitamin A. In wheat, Velu et al. (83) reported genome-wide predictions for grain Fe and Zn concentrations in a diverse panel of 330 genotypes with prediction accuracies ranging from 0.331 to 0.694 and 0.324 to 0.734, respectively. Another study in wheat also found moderate to high genomic prediction accuracies for various major and minor elements concentration in grains (85). The high genomic prediction accuracies for mineral nutrient traits suggest that GS holds great potential in accelerating breeding for biofortification traits. Further, speed breeding that enables taking up to six generations in one year under glasshouse can be very well combined with GS in different breeding schemes to accelerate genetic gain for biofortification traits (Figure 1).

## A Brief Account of Biofortified Wheat Varieties Developed Through Conventional Breeding

In addition to basic research on micronutrients acquisition, the development of biofortified wheat varieties has recently upscaled with several successful examples. The conventional breeding approach has demonstrated great potential to biofortify hexaploid wheat genotypes by identifying suitable donor genetic resources such as synthetic, wild and primitive wheat genotypes for high Fe and Zn content with enhanced bioavailability. The most promising high Zn and Fe sources are diploid progenitors of hexaploid wheat (*Aegilops tauschii)*, wild emmer (*T. dicoccoides*), einkorn (*Triticum monococcum*), *T. spelta*, *T. polonicum*, and *T. aestivum* landraces. Among wild wheat tested so far, the collections of wild emmer wheat, *Triticum turgidum* ssp. *dicoccoides*, showed a prominent genetic variation of Zn ranging from 14 to 190 mg kg^–1^ and Fe up to 88 mg kg^–1^ (13). Translocation from different *Aegilops spp.* and rye to Pavon 76 background at the International Maize and Wheat Improvement Center (CIMMYT, Mexico) has generated several synthetic hexaploids (SHW), *T. spelta*, and several pre-breeding lines having wider variation in Zn (38 to 72 mg kg^–1^) and Fe content (32 to 52 mg kg^–1^) (34).

With these concerted efforts, CIMMYT wheat breeders, in collaboration with other major institutions of India, Pakistan, Bangladesh, Nepal, and Bolivia, have facilitated the development and release of 40 biofortified wheat varieties for commercial cultivation (Table 2). Since 2014, A total of 24 biofortified wheat varieties (*T. aestivum*; 16 and *T. durum*; 8) for Fe, Zn and protein have been developed by ICAR-Indian Institute of Wheat and Barley Research, Karnal, Punjab Agricultural University, Ludhiana, ICAR- Indian Agricultural Research Institute, Delhi, Agharkar Research Institute, Pune, University of Agricultural Sciences, Dharwad, Banaras Hindu University, Varanasi and private seed companies and released to Indian farmers for common cultivation at different wheat growing zones of India. Similarly, CIMMYT, in collaboration with important wheat research institutions of Pakistan, Bangladesh, Bolivia, Nepal and Mexico, has developed and released 2, 1, 1, 2, and 1 biofortified wheat varieties, respectively (Table 2). Overall, utilization of wild relatives and SHW of wheat in conventional breeding programs have significantly impacted the development of micronutrient biofortified wheat varieties, which is expected to continue with increased bioavailability. Over the next two decades, developing and mainstreaming Zn and Fe in the wheat breeding program at CIMMYT and partner institutions across the globe would undoubtedly enable the release of high-yielding and Fe and Zn biofortified wheat varieties to a more significant percentage of farmers of South Asia to curb hidden hunger of children and pregnant and lactating mothers.

**TABLE 2:** List of biofortified wheat varieties developed through conventional breeding and released for commercial cultivation around the globe.

Variety	Nutritional quality	Year of release	Developer/sources
**India**			
DDW 48 (*T. durum*)	Fe: 38.8; Zn: 39.7; Protein: 12.1	2020	ICAR-Indian Institute of Wheat and Barley Research, Karnal, India
DDW 47 (*T. durum*)	Fe: 40.1; Protein: 12.7	2020	
DBW 303	Fe: 35.8; Zn: 36.9; Protein: 12.1	2020	
DBW 187	Fe: 43.1	2018 and 2020	
DBW 173	Fe: 40.7; Protein: 12.5	2018	
WB 02	Zn: 42; Fe: 40	2017	
PBW 771	Zn: 41.4	2020	Punjab Agricultural University (PAU), Ludhiana, India
PBW 752	Fe: 37.1; Zn: 38.7; Protein: 12.4	2018	
PBW 757	Zn: 42.3	2018	
HPBW 01	Zn: 40.6; Fe: 40	2017	
HI 8802 (*T. durum*)	Fe: 39.5; Zn: 35.9; Protein: 13.0	2020	ICAR- Indian Agricultural Research Institute, Regional Station, Indore, India
HI 8805 (*T. durum*)	Fe: 40.4; Protein: 12.8	2020	
HI 1633	Fe: 41.6; Zn: 41.1; Protein: 12.4	2020	
HI 8759 (*T. durum*)	Zn: 42.8; Fe: 42.1; Protein: 12.0	2017	
HI 1605	Zn: 35; Fe: 43; Protein: 13	2017	
HI 8777 (*T. durum*)	Fe: 48.7; Zn: 43.6	2017	
HD 3171	Zn: 47.1	2017	ICAR- Indian Agricultural Research Institute, New Delhi, India
HD 3249	Fe: 42.5	2020	
HD 3298	Fe: 43.1; Protein:12.1	2020	
MACS 4028 (*T. durum*)	Zn: 40.3; Fe: 46.1; Protein: 14.7	2018	Developed by Agharkar Research Institute, Pune, Maharashtra
MACS 4058 (*T. durum*)	Fe: 39.5 Zn: 37.8 Protein: 14.7	2020	
UAS 375	Protein: 13.8	2018	University of Agricultural Sciences, Dharwad, India
BHU-3	High Zn	2014	Banaras Hindu University, Varanasi, India
Abhay	High Zn	2015	Nirmal Seeds, Harvest Plus and Participatory variety selection
Chitra	High Zn	2016	Participatory variety selection
**Pakistan**			
NR- 421 (Zincol-16)	High Zn (> 6 ppm Zn advantage compared to best local check)	2015	Pakistan Agriculture Research Council/CIMMYT
Akbar-19	High Zn (> 7 ppm Zn advantage compared to best local check)	2019	Faisalabad Agricultural Research Institute/CIMMYT
**Bangladesh**			
BARI Gom 33	High Zn (7–8 ppm Zn advantage over best check, and also resistance to wheat blast)	2017	CIMMYT, Mexico
**Mexico**			
Nohely-F2018	High Zn (released in Mexico for the Mexicali valley of northern Sonora region)	2018	CIMMYT, Mexico
**Bolivia**			
Iniaf-Okinawa	High Zn (> 6 ppm Zn advantage than the local check)	2018	INIAF, Bolivia and CIMMYT, Mexico
**Nepal**			
Zinc Gahun 1	High Zn (> 6 ppm Zn advantage than the local check)	2020	NARC, Nepal and CIMMYT, Mexico
Zinc Gahun 2			

*Grain Fe and Zn conents are expressed in ppm while protein content is expressed in percentage (%).*

## Genetically Modified Biofortified Wheat

**FIGURE 2:**
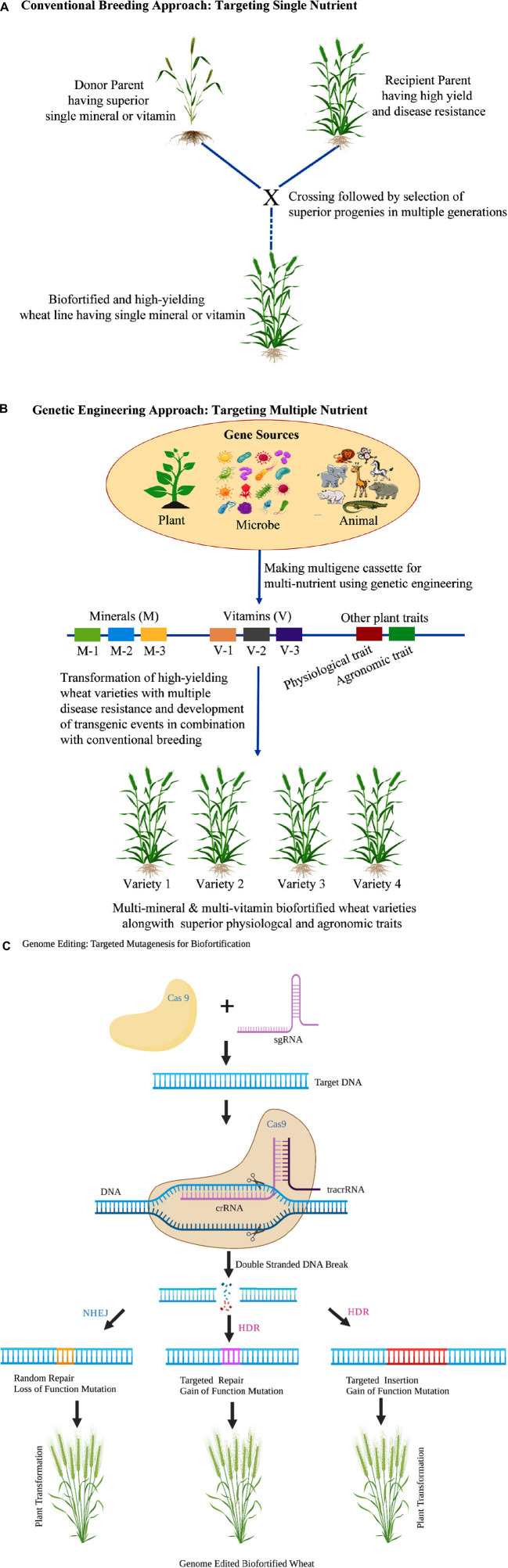
Schematic representation of conventional **(A)** genetic engineering **(B)** and genome editing **(C)** approaches in wheat for targeted biofortifcation of micronutrients. Genetic engineering and genome editing approaches in combination with conventional breeding offers simultaneous incorporation of multi-nutrient (minerals and vitamins) traits along with improved physiological and agronomic features.

While conventional breeding is globally accepted, the absence of desired genetic diversity within the primary, secondary and tertiary gene pools for targeted traits within species (e.g., golden rice) or difficult to breed crops (e.g., banana) can efficiently be managed through genetic engineering technologies as a viable alternative. Also, the development of multi-nutrient cultivars by stacking multiple genes coupled with superior physiological and agronomic traits is often limited with conventional breeding, which can be circumvented by the genetic engineering approach (Figures 2A,B). However, wheat being hexaploid is comparatively challenging to transform and therefore needs the development of a robust transformation protocol to harness the full potential of the transgenic approach. As demonstrated in Figure 2B, the genetic engineering approach offers limitless cross-kingdom utilization and tacking of desired genes for multi-nutrients target traits improvement, including biotic and abiotic stresses, making it more attractive for farmers to adopt nutritionally improved nutrition wheat varieties. Moreover, it offers simultaneous biofortification of multi nutrients by metabolic engineering (86). However, the three significant bottlenecks of the transgenic approach are the lack of availability of suitable transformation protocol in polyploidy crop such wheat, fear of environmental escape of transgene and global genetically modified organisms (GMO) regulation. Knowledge gained in identifying and functional characterization of different genes actively associated with uptake and translocation of Fe and Zn can efficiently be used to increase Fe and Zn content in wheat by transgenic approach. Several proofs of concepts using the genetic engineering approaches have been tested with apparently stirring results in wheat for grain Fe and Zn. For example, The *NAM-B1(Gpc-B1)* transcription factor provides an entry point to increase Fe and Zn content. Knowing the critical control points, we can modify expression patterns, downstream targets or binding specificities to augment micronutrient content in grains. Wheat biofortification for Fe and Zn has been achieved using the transcription factor *NAM-B1* (24), which was initially identified for increasing protein content in wild emmer (*Triticum turgidum* ssp *dicoccoides*). In recombinant substitution lines (RSL), the presence of *NAM-B1* allele of *T. dicoccoides* increased Fe and Zn grain concentrations by 18 and 12%, respectively, in addition to 38% higher protein as compared with RSLs carrying the allele from cultivated wheat (*Triticum durum*) (87). Further, the increase in grain Fe and Zn content did not significantly correlate with yield reduction across the five environments (87). This gene is being widely used in breeding programs across several continents (88, 89).

Transgenic approaches involving endosperm-specific expression of wheat ferritin, *TaFer1-A* (90) or soybean ferritin (91) led to 1.5- to 1.9-fold and 1.1- to 1.6-fold increase in grain Fe, respectively alongside increased phytase activity (92). However, the stability of wheat *TaFer1-A* in subsequent generations remains a question. Two independent workers have depicted that the overexpression of *NICOTIANAMINE SYNTHASE 2* (*OsNAS2*) gene in wheat produced Fe up to 93.1 mg g^–1^ (93) and 80 mg g^–1^ (94) under greenhouse and field conditions, respectively. Connorton et al. (95) demonstrated the doubling of total Fe content in wheat flour by using *VACUOLAR IRON TRANSPORTER 2* (*TaVIT2*) gene, which effectively enhances vacuolar Fe and manganese (Mn) transport in the endosperm. In addition to micronutrients, progress has been made to discourse the challenges of most deficient nutrients like vitamin A and quality proteins in wheat. The provitamin A content of wheat has been enhanced by expressing bacterial *Phytoene synthase (CrtB)* and *Carotene desaturase* gene (*CrtI)* (96, 97). To increase Fe bioavailability, phytase activity was increased by expressing the *Phytochrome* (*phyA)* gene (98), while phytic acid content was decreased by silencing the wheat *ABCC13* transporter gene (99). Protein content, especially essential amino acids lysine, methionine, cysteine, and tyrosine contents in wheat grains, were also attempted to enhance using Amaranthus albumin gene *ama1* (100). Wheat has also been targeted to improve the antioxidant activity by expressing maize regulatory genes *C1*, *B-peru* involved in anthocyanin production (101). The development of biofortified crop varieties either by conventional breeding or transgenic methods is considered a sustainable solution to the problem of micronutrient deficiency. The advantage of this strategy over others like dietary diversification, food fortification, and food supplementation is that once the initial research and development is completed, the benefits of the nutritionally enhanced crops will be sustainable with little further investment. With the advent of powerful reverse genome editing tools such as transcription activator-like effector nucleases (TALENs) and Clustered regularly interspaced short palindromic repeats/CRISPR associated protein 9 (CRISPR/Cas9) coupled with fully sequenced genomes of wheat can be tested as a proof-of-concept for multiple micronutrients biofortification by targeting genes associated with micronutrients uptake and redistribution in different tissues. This will increase the biochemical and physiological pathway’s efficiency system biology (pathway reconstruction) and decrease the anti-nutritional factor to increase the bioavailability.

## Utilizing Natural Genetic Variation for Improving Nutritional Quality Through Genome Editing Technologies

Genome editing technologies have emerged as advanced biotechnological, new plant breeding techniques, which offer efficient, target-specific and accurate approaches to engineer genome of a plant. The recent development of CRISPR/Cas9 based genome editing technologies in wheat has shown a ray of hope for improving the nutritional quality of wheat grains (Figure 2C). Edited gene constructs generated by these techniques have been delivered to the host genome by PEG-mediated protoplast fusion, particle gun bombardment, or ribonucleoprotein complex. *Since wheat carries a complex hexaploid genome, CRISPR/Cas9—mediated* wheat geminiviral based DNA replicons, delivering RNPs by biolistic method and multiplex editing has proved to be a more appropriate method of genome editing (102, 103). The TALEN and CRISPR/Cas9 systems have already demonstrated their utility for generating abiotic and biotic stress-resistant engineered wheat plants (37). For instance, the mildew resistance locus O (*TaMLO*) gene was edited by CRISPR/Cas9 and TALEN through PEG-mediated protoplast fusion method (39, 104), to achieve resistance to the fungal pathogen. Further, CRISPR/Cas9 genome editing system was applied to engineer dehydration responsive element-binding protein 2 (*TaDREB2*) and wheat ethylene-responsive factor 3 (*TaERF3*) to increase abiotic stress tolerance (105). A more sophisticated technique of multiplexed genome editing with CRISPR/Cas9 has also been demonstrated for wheat using *TaGW, TaLpx* and *TaMLO* genes (103). The gene editing approach has been deployed to improve wheat’s grain traits by utilizing the CRISPR/Cas9 RNP delivery of *TaGW2* and *TaGASR7* (negative regulators of grain traits and kernel weight) for increasing kernel weight (102). However, some recent studies have highlighted the use of genome editing for breeding varieties with improved grain quality and increased nutritional value in wheat. CRISPR/Cas9 system was applied to obtain a wheat variety with hypoimmunogenic gluten content by editing α-gliadin genes (40, 106). Similarly, high-amylose modern wheat varieties, needed for better human health, were developed through targeted mutagenesis of the gene *TaSBEIIa* by CRISPR/Cas 9 system (107). Further, the CRISPR/Cas9 editing tool has been demonstrated to be effective in simultaneous editing of multiple genes such as large α- and γ-gliadin gene families in the polyploid bread wheat (106). Other grain quality characteristics such as hardness, starch composition and dough color have been altered in wheat by targeting the *pinb*, *waxy*, *ppo* and *psy* genes (108).

Application of gene-editing techniques is required to be utilized furthermore for biofortification of wheat for enhancing Fe, Zn, Se, Ca and other micronutrient contents. Through QTL mapping and association mapping, information on genomic regions/QTLs responsible for grain Zn and Fe concentrations in different wheat varieties is now available, which can enhance Fe and Zn content in the high yielding varieties. Transgenic technology has been used to generate genetically modified plants with enhanced Fe content as well as better absorption by deploying different genes such as *NAM-B1* transcription factor gene (24), *TaFer1-A* (90, 91), *NAS2* (93, 94), *TaVIT2* (95), and *Phytochrome* (phyA) (98). However, it is recommended that genome editing by CRISPR/Cas9 strategy be applied to provide marker- and foreign DNA- free genetically engineered plants with high Fe content and increased absorption. Attempts are needed to identify and validate genomic regions/QTLs contributing to phytic acid level in wheat grain which can then be engineered to modulate its level by the gene-editing system. For instance, *ABCC13* can be knocked out to reduce the quantity of phytic acid to enhance the concentration of bioavailable minerals. Similarly, future research should focus on enhancing essential amino acid concentration and vitamin levels in wheat. Moreover, *CRISPR/cas9—based genome editing systems can also be utilized to trim the unwanted sequences like marker gene, T-DNA region, etc. from the transgenic plants. It is highly recommended that the genome-edited wheat genotypes with improved grain quality characteristics be deployed regularly in wheat breeding programs to enrich the agronomically superior varieties with high nutritional value.* It is also envisioned that more recently developed techniques like precise genome editing through base editors, and prime editors are utilized to improve grain quality efficiently and effectively (38, 109).

## Conclusion and Future Prospects

Wheat is the most widely cultivated, prominent food crop. There have been several attempts to improve wheat quality and crop yield after the “green revolution”. However, the main focus of wheat improvement programs has been on high yield, resulting in high yielding wheat varieties over time but with suboptimal levels of minerals and micronutrients. In order to achieve food and nutritional security, and to provide an adequate supply of calories and nutrients, it is imperative to improve both qualitative and quantitative traits of wheat. Biofortification of wheat can be achieved by exploring natural genetic diversity in wheat and its wild species for higher minerals and phytonutrients through utilizing advanced genomic tools such as QTL mapping and genome-wide association study (GWAS) for mapping nutritional quality traits in wheat, molecular breeding approaches, genomic selection, and genome engineering by transgenic technology and genome editing strategies. Genetic diversity studies have been performed, and few wild relatives of wheat, *T. dicoccoides, Aegilops tauschii, T. dicoccoides*, *T. boeoticun*, *T. spelta*, *T. polonicum*, and *T. aestivum* landraces have been identified that carry relatively higher concentrations of Fe, Zn and Mn. Similarly, QTL mapping and GWAS studies on wheat led to identifying loci responsible for grain Zn and Fe concentrations. Many attempts have been made using transgenic technology to generate wheat with better mineral and micronutrient content and transgenic wheat lines with higher content of Fe, Mn and Vit A have been reported. However, more recently, genome engineering tools like gene editing *via* CRISPR/Cas system, prime editors and base editors have gained much more popularity among scientists over conventional breeding and transgenic technology because of their efficacy, precision, simplicity and robustness.

The significant advantage of genome editing is that it eliminates the foreign DNA/transgene from the final engineered plants. Genome editing by TALEN and CRISPR/Cas 9 system has been employed in wheat to improve stress tolerance and grain yield; however, this system has not been explored as much for biofortification of wheat. Therefore, considering that biofortification of wheat is essential for improving grain quality, genome editing needs to be deployed to improve the content of Fe, Zn, Se, Ca, essential amino acids and decrease the concentration of antinutrients such as phytic acid. Other methods such as multiplex gene editing, transiently expressing CRISPR/Cas9, base editing, prime editing and CRISPR/Cas9 ribonucleoproteins are also promising and should be considered for future research. Exploring natural genetic diversity and broadening the narrow genetic base of hexaploid cultivated wheat varieties is essential and warrants greater attention through whole-genome sequencing of large number of accessions (e.g., the composite core set), and functional genomics for gene discovery associated with agronomic and nutritional traits (110, 111). Such efforts could help generate useful genetic information and genomic resources for accelerating wheat improvement through genome editing (112). Gene editing in germline cells and the CRISPR system carrying RNA interference elements need to be explored in wheat. Similarly, epigenetic genome modifications deserve attention. Also, simulation model-based prediction of superior wheat quality traits under different environmental conditions (113) might accelerate the global wheat nutritional quality program. Hence, utilization of these techniques to improve the nutritional quality of wheat grains and combine them with high yielding traits is emphasized.

## Author Contributions

OPG, AKS, GPS, KCB, and SKD conceived the idea and designed the outline. OPG, AKS, AS, and KCB drafted the manuscript. OPG, AKS, and AS prepared the illustrations. KCB, GPS, and SKD reviewed and improved the draft. All authors collected, compiled, analyzed, and interpreted the literature and contributed significantly to the article and approved the final version.

## Conflict of Interest

The authors declare that the research was conducted in the absence of any commercial or financial relationships that could be construed as a potential conflict of interest.

## Publisher’s Note

All claims expressed in this article are solely those of the authors and do not necessarily represent those of their affiliated organizations, or those of the publisher, the editors and the reviewers. Any product that may be evaluated in this article, or claim that may be made by its manufacturer, is not guaranteed or endorsed by the publisher.

## Footnotes

1
https://genomics.cimmyt.org/
